# Mode of Diagnosing Buffy Blood

**Published:** 1846-10

**Authors:** 


11. *Mode of Diagnosing Buffy Blood.*—Dr. Wharton Jones has pointed out a very ingenious method of determining whether the blood is buffy or not, from an examination of a very minute portion of this fluid. It consists of quickly enclosing a drop between two pieces of glass, and observing, with the naked eye, the quickness with which it assumes a mottled appearance, and the smallness or largeness of the interspaces. In buffy blood the mottling is almost instantaneous, and the interspaces large; while in healthy blood it is delayed for half a minute or more, and the reticulation is minute.—*Dr. Cowan's Address*, in *Southern Medical Journal.*

